# Socioeconomic Disparities in Six Common Cancer Survival Rates in South Korea: Population-Wide Retrospective Cohort Study

**DOI:** 10.2196/55011

**Published:** 2024-07-22

**Authors:** JinWook Lee, JuWon Park, Nayeon Kim, Fatima Nari, Seowoo Bae, Hyeon Ji Lee, Mingyu Lee, Jae Kwan Jun, Kui Son Choi, Mina Suh

**Affiliations:** 1 National Cancer Control Institute, National Cancer Center, Goyang, Republic of Korea; 2 Department of Public Health, Graduate School, Yonsei University, Seoul, Republic of Korea; 3 Graduate School of Cancer Science and Policy, National Cancer Center, Goyang, Republic of Korea

**Keywords:** cancer survival, income level, socioeconomic status, deprivation index, inequality, nationwide analysis, cancer, South Korea, public health

## Abstract

**Background:**

In South Korea, the cancer incidence rate has increased by 56.5% from 2001 to 2021. Nevertheless, the 5-year cancer survival rate from 2017 to 2021 increased by 17.9% compared with that from 2001 to 2005. Cancer survival rates tend to decline with lower socioeconomic status, and variations exist in the survival rates among different cancer types. Analyzing socioeconomic patterns in the survival of patients with cancer can help identify high-risk groups and ensure that they benefit from interventions.

**Objective:**

The aim of this study was to analyze differences in survival rates among patients diagnosed with six types of cancer—stomach, colorectal, liver, breast, cervical, and lung cancers—based on socioeconomic status using Korean nationwide data.

**Methods:**

This study used the Korea Central Cancer Registry database linked to the National Health Information Database to follow up with patients diagnosed with cancer between 2014 and 2018 until December 31, 2021. Kaplan-Meier curves stratified by income status were generated, and log-rank tests were conducted for each cancer type to assess statistical significance. Hazard ratios with 95% CIs for any cause of overall survival were calculated using Cox proportional hazards regression models with the time since diagnosis.

**Results:**

The survival rates for the six different types of cancer were as follows: stomach cancer, 69.6% (96,404/138,462); colorectal cancer, 66.6% (83,406/125,156); liver cancer, 33.7% (23,860/70,712); lung cancer, 30.4% (33,203/109,116); breast cancer, 91.5% (90,730/99,159); and cervical cancer, 78% (12,930/16,580). When comparing the medical aid group to the highest income group, the hazard ratios were 1.72 (95% CI 1.66‐1.79) for stomach cancer, 1.60 (95% CI 1.54‐1.56) for colorectal cancer, 1.51 (95% CI 1.45‐1.56) for liver cancer, 1.56 (95% CI 1.51‐1.59) for lung cancer, 2.19 (95% CI 2.01‐2.38) for breast cancer, and 1.65 (95% CI 1.46‐1.87) for cervical cancer. A higher deprivation index and advanced diagnostic stage were associated with an increased risk of mortality.

**Conclusions:**

Socioeconomic status significantly mediates disparities in cancer survival in several cancer types. This effect is particularly pronounced in less fatal cancers such as breast cancer. Therefore, considering the type of cancer and socioeconomic factors, social and medical interventions such as early cancer detection and appropriate treatment are necessary for vulnerable populations.

## Introduction

Cancer is a major global cause of disease burden and one of the top three causes of death [1]. In South Korea, cancer is the leading cause of death [2], with 277,523 new cases of cancer reported in 2021 [3]. The most common cancer sites were the thyroid, lung, colon and rectum, stomach, and breast [3]. Among these, lung cancer has the highest mortality rate [2]. The South Korean government has established the National Cancer Screening Program (NCSP) to provide organized cancer screening to alleviate the burden of cancer [4], and the NCSP has contributed to a reduction in cancer mortality rates [5-8] The cancer incidence rate has increased by 56.5% from 2001 to 2021 [2]. Nevertheless, recent data from the past 5 years (2017‐2021) have shown a 17.9% increase in the 5-year survival rate for diagnosed patients with cancer compared with those diagnosed between 2001 and 2005 [9].

Cancer survival rates decrease with lower socioeconomic status (SES) factors such as income and educational levels [10-17]. Furthermore, the association between cancer incidence and mortality rates and SES varies according to cancer type [18-22]. Notably, for relatively preventable cancers such as cervical cancer, the mortality rate in the low-income group was twice that of the high-income group, whereas disparities in mortality rates for lung and liver cancers were comparatively narrower [11]. Furthermore, studies from Japan and the United States have found a correlation between measuring SES levels using the regional deprivation index and the risk of cancer mortality [23,24]. However, a similar relationship was not identified in a Korean study [25]. Therefore, more recent assessments are required to reduce socioeconomic disparities among patients with cancer.

In studies related to cancer survival rates, the stage of diagnosis is a significant factor explaining survival disparities [12,26-28]. However, despite extensive studies, the relationship between SES and disease stage at diagnosis and its effects on survival remains inconclusive. Studies in the United States have found an association between SES and disease stage at diagnosis [26,29], but a large-scale study conducted in the United Kingdom did not confirm these observations, despite noting survival disparities among social groups [30].

Analyzing socioeconomic patterns in the survival of patients with cancer is important because it allows the quantification of cancer-related health disparities between the most disadvantaged and most advantaged social groups [18]. These analyses help identify regions or population groups at the highest risk of death so that they can benefit from interventions such as early cancer screening and appropriate cancer treatment [18,20].

In many countries, large-population data have been utilized to analyze differences in cancer survival rates based on SES across various cancer types [11,18-20,31]. However, studies from this perspective have been limited to South Korea. Therefore, this study aims to analyze differences in survival rates among patients diagnosed with six types of cancer—stomach, colorectal, liver, breast, cervical, and lung cancers—based on SES using Korean nationwide data.

## Methods

### Data Source and Study Population

The Korean National Health Insurance Service (NHIS) is a government insurer that promotes the health of the population by providing a universal health insurance program that covers the entire population. The National Health Information Database (NHID) of the NHIS is a public database of anonymized participant information on sociodemographic variables, medical records, health care use, health screening, and mortality across the entire population. Further details regarding the database profile have been outlined elsewhere [32,33].

Our study data were obtained from the Korea Central Cancer Registry (KCCR) database, which was then linked to the national claims data from the NHID using unique personal identification numbers with the consent of the KCCR (NHIS-2022-1-114) [32,34]. The included populations in this analysis were patients who were primarily diagnosed with stomach cancer (C16), colorectal cancer (C18-20), liver cancer (C22), lung cancer (C33-34), breast cancer (C50), and cervical cancer (C53) based on *International Statistical Classification of Diseases, Tenth Revision* codes from 2014 to 2018 and were followed up with until December 31, 2021 [35].

In total, 11,209 patients with unknown income status, regional area that indicates the deprivation index quartile, and survival period were excluded for gastric cancer (n=140,873), colon cancer (n=127,525), liver cancer (n=72,303), lung cancer (n=111,284), breast cancer (n=101,752), and cervical cancer (n=17,032). This study analyzed the survival rates of the remaining patients with stomach cancer (n=138,462), colorectal cancer (n=125,156), liver cancer (n=70,712), lung cancer (n=109,116), breast cancer (n=99,553), and cervical cancer (n=16,580).

### Ethical Considerations

This study was approved by the institutional review board of the National Cancer Center (NCC2021-0264). The review board waived the requirement for written informed consent from the patients because the data are public and anonymized under confidentiality guidelines.

### Definition of Variables

Income level was classified into six categories. After being classified as National Medical Aid beneficiaries or National Health Insurance subscribers (for workplace health insurance subscribers and local subscribers), the latter group was subclassified into five segments, from the lowest (first, grades 1‐4) to highest (fifth, grades 17‐20), according to 20 insurance premium grades. The National Medical Aid program is a form of subsidized government assistance that provides coverage to low-income populations with health care services [36].

The Korean deprivation index, developed by the Korea Institute for Health and Social Affairs, is an indicator of the SES of a region by considering nine socioeconomic determinants: living alone, not owning a car, having an underdeveloped living environment, not living in an apartment, living in a female-headed household, having a low level of education, having an older population, having low social class, and being divorced or widowed [37]. Higher deprivation index values indicate more significant levels of deprivation, with values spanning from −20.33 to 15.15. Korea comprises 250 municipal-level units (otherwise known as *sigungu*), and deprivation and cancer survival rates were provided for all municipal-level areas [38]. The deprivation index was further divided into quartiles, with Q1 representing the most deprived and Q4 indicating the least deprived. In this categorization, the first quartile included 25% of the municipal-level units with the lowest deprivation scores, whereas the fourth quartile encompassed 25% of the municipal-level units with the highest deprivation scores.

Age at diagnosis was classified into three groups: ≤49, 50‐69, and ≥70 years. The stage at diagnosis was classified as localized, regional, distant, and unknown using the Surveillance, Epidemiology, and End Results (SEER) staging system.

### Statistical Analyses

Kaplan-Meier curves stratified by income status were generated, and log-rank statistical significance tests were performed for each cancer type. Hazard ratios (HRs) with 95% CIs for any cause of overall survival were calculated using Cox proportional hazards regression models with time since diagnosis. The model was adjusted for sex, age at diagnosis, stage at diagnosis, and deprivation index. Income status was the primary predictor after adjusting for other statistically significant variables in the Kaplan-Meier survival log-rank tests.

SAS version 9.4 (SAS Institute) was used for all analyses, and the figures were generated using R software version 4.3.0 (The R Foundation for Statistical Computing). *P* values <.05 were considered statistically significant.

## Results

**Figure 1.**
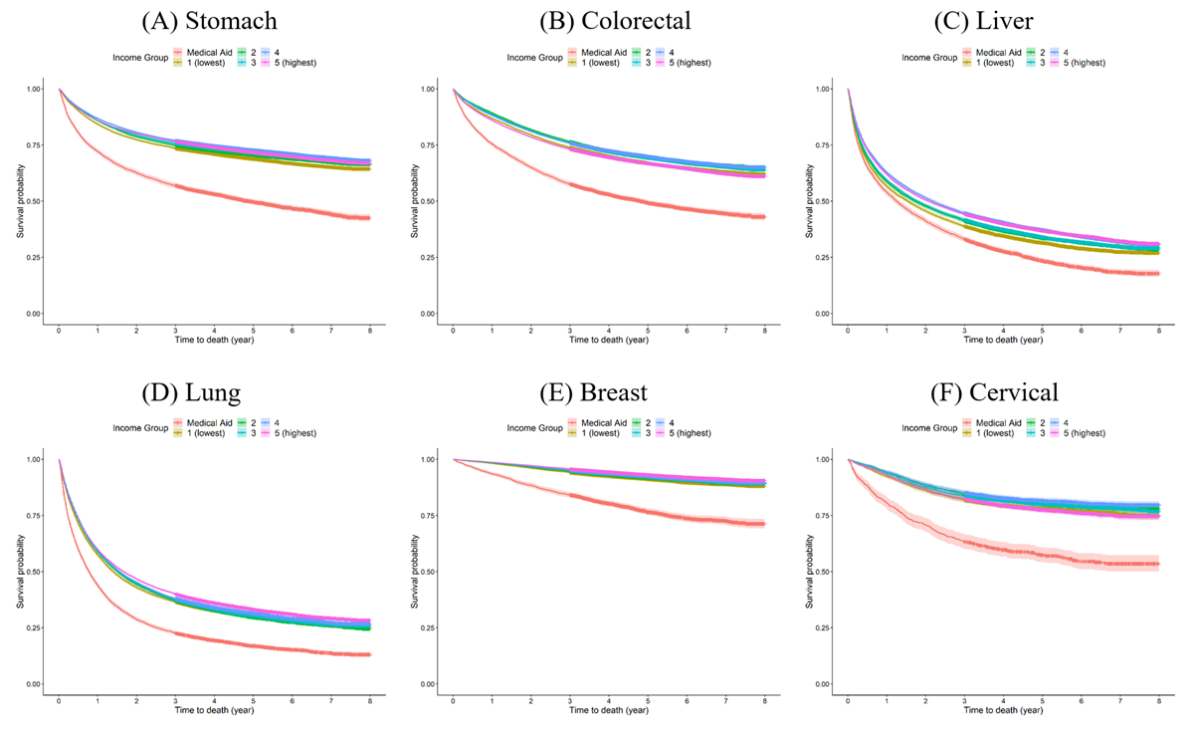
Kaplan-Meier curves for (A) stomach, (B) colorectal, (C) liver, (D) lung, (E) breast, and (F) cervical cancers among different income groups.

Table 1 provides an overview of the general characteristics of the patients with cancer. Across the income groups, the highest income group had the highest proportion of all six cancer types. The proportions were as follows: stomach cancer, 30.2% (41,792/138,462); colorectal cancer, 28.5% (35,663/125,156); liver cancer, 26.8% (18,976/70,712); lung cancer, 31.3% (34,159/109,116); breast cancer, 28.2% (27,929/199,159); and cervical cancer, 20.5% (3403/16,580). The medical aid group had the lowest proportions: 4.8% (6633/138,462) for stomach cancer, 5.7% (7160/125,156) for colorectal cancer, 7.1% (4989/70,712) for liver cancer, 7% (7650/109,116) for lung cancer, 3.3% (3305/99,159) for breast cancer, and 5.3% (887/16,580) for cervical cancer. The distribution of SEER stages was as follows: stomach cancer, 63.7% (88,263/138,462); liver cancer, 45.4% (32,136/70,712); breast cancer, 58.4% (57,891/99,159); and cervical cancer, 55.4% (9187/16,580), indicating the highest proportion of localized cases. However, for colorectal cancer, 42.4% (53,004/125,156) of cases were in the regional stage, whereas lung cancer had the highest proportion in the distant stage (47,789/109,116, 43.8%). The deprivation index revealed that for all six cancer types, the highest proportion was in Q4 (least deprived) and the lowest proportion was in Q1 (most deprived). The survival rates for stomach, colorectal, liver, lung, breast, and cervical cancers were 69.6% (96,404/138,462), 66.6% (83,406/125,156), 33.7% (23,860/70,712), 30.4% (33,203/109,116), 91.5% (90,730/99,159), and 78% (12,930/16,580), respectively. Breast cancer had the highest survival rate, whereas lung cancer had the lowest. The mean survival times for stomach, colorectal, liver, lung, breast, and cervical cancers were 5.84 (SD 3.01), 5.73 (SD 3.01), 3.35 (SD 3.30), 3.09 (SD 3.20), 7.17 (SD 1.60), and 6.38 (SD 2.55) years, respectively. The Kaplan-Meier analysis and log-rank test revealed significant differences in survival rates among the income level groups (Figure 1).

**Table 1.** General characteristics of patients with cancer.

Variable		Stomach(n=138,462)		Colorectal(n=125,156)		Liver (n=70,712)		Lung(n=109,116)		Breast(n=99,159)		Cervical (n=16,580)	
		Patients, n	%	Patients, n	%	Patients, n	%	Patients, n	%	Patients, n	%	Patients, n	%
**Sex**													
	Male	93,240	67.3	74,086	59.2	52,846	74.7	75,225	68.9	0	0.0	0	0.0
	Female	45,222	32.7	51,070	40.8	17,866	25.3	33,891	31.1	99,159	100.0	16,580	100.0
**Age at diagnosis (years)**													
	≤49	17,620	12.7	14,376	11.5	8671	12.3	5358	4.9	43,814	44.2	7689	46.4
	50‐69	70,373	50.8	60,160	48.1	37,869	53.6	46,898	43.0	45,979	46.4	6120	36.9
	≥70	50,469	36.4	50,620	40.4	24,172	34.2	56,860	52.1	9366	9.5	2771	16.7
**Surveillance, Epidemiology, and End Results stage**													
	Localized	88,263	63.7	44,357	35.4	32,136	45.4	24,275	22.2	57,894	58.4	9187	55.4
	Regional	28,001	20.2	53,004	42.4	17,440	24.7	28,535	26.2	33,297	33.6	4717	28.4
	Distant	15,246	11.0	20,782	16.6	10,992	15.5	47,789	43.8	4911	5.0	1531	9.2
	Unknown	6952	5.0	7013	5.6	10,144	14.3	8517	7.8	3060	3.1	1145	6.9
**Income**													
	Medical aid	6633	4.8	7160	5.7	4989	7.1	7650	7.0	3305	3.3	887	5.3
	1 (lowest)	20,694	14.9	19,825	15.8	10,977	15.5	16,010	14.7	16,911	17.1	3249	19.6
	2	18,883	13.6	17,395	13.9	10,239	14.5	13,563	12.4	15,203	15.3	2872	17.3
	3	21,564	15.6	19,883	15.9	11,443	16.2	16,014	14.7	15,832	16.0	3031	18.3
	4	28,896	20.9	25,230	20.2	14,088	19.9	21,720	19.9	19,979	20.2	3138	18.9
	5 (highest)	41,792	30.2	35,663	28.5	18,976	26.8	34,159	31.3	27,929	28.2	3403	20.5
**Deprivation index**													
	Q1 (most deprived)	14,948	10.8	11,985	9.6	7975	11.3	13,107	12.0	4798	4.8	1136	6.9
	Q2	26,606	19.2	23,767	19.0	14,150	20.0	21,647	19.8	15,155	15.3	2904	17.5
	Q3	48,845	35.3	45,537	36.4	25,176	35.6	38,355	35.2	36,469	36.8	6223	37.5
	Q4 (least deprived)	48,063	34.7	43,867	35.0	23,411	33.1	36,007	33.0	42,737	43.1	6317	38.1

After adjusting for sex, age at diagnosis, SEER stage, and deprivation index, the risk of death increased as the income level decreased across all six cancer types (Table 2). When comparing the medical aid group to the highest income group, the HRs were 1.72 (95% CI 1.66‐1.79) for stomach cancer, 1.60 (95% CI 1.54‐1.56) for colorectal cancer, 1.51 (95% CI 1.45‐1.56) for liver cancer, 1.56 (95% CI 1.51‐1.59) for lung cancer, 2.19 (95% CI 2.01‐2.38) for breast cancer, and 1.65 (95% CI 1.46‐1.87) for cervical cancer. A high deprivation index was associated with a higher risk of death, especially when comparing the least deprived group with the most deprived group. In these cases, the HRs for stomach, colorectal, liver, lung, breast, and cervical cancers were 1.13 (95% CI 1.10‐1.17), 1.13 (95% CI 1.09‐1.17), 1.08 (95% CI 1.05‐1.12), 1.19 (95% CI 1.16‐1.22), 1.19 (95% CI 1.09‐1.31), and 1.16 (95% CI 1.03‐1.31), respectively. Furthermore, as the disease progressed and the patients aged, a higher risk of death was observed.

**Table 2.** Adjusted hazard ratios (HRs) from Cox proportional hazards regression model among patients with cancer. Adjusted for sex; age at diagnosis; income; Surveillance, Epidemiology, and End Results stage; and deprivation index.

Variable		Stomach		Colorectal		Liver		Lung		Breast		Cervical	
		HR	95% CI	HR	95% CI	HR	95% CI	HR	95% CI	HR	95% CI	HR	95% CI
**Sex**													
	Male	1.00	Reference	1.00	Reference	1.00	Reference	1.00	Reference	—^a^	—	—	—
	Female	0.93	0.91‐0.95	0.92	0.91‐0.94	0.94	0.92‐0.96	0.60	0.59‐0.61	—	—	—	—
**Age at diagnosis (years)**													
	≤49	1.00	Reference	1.00	Reference	1.00	Reference	1.00	Reference	1.00	Reference	1.00	Reference
	50‐69	1.15	1.11‐1.19	1.31	1.26‐1.37	1.04	1.01‐1.07	1.43	1.37‐1.49	1.30	1.23‐1.36	1.16	1.06‐1.26
	≥70	3.14	3.03‐3.26	3.96	3.81‐4.13	2.00	1.93‐2.06	3.10	2.97‐3.23	4.32	4.07‐4.58	3.90	3.58‐4.24
**Income**													
	Medical aid	1.72	1.66‐1.79	1.60	1.54‐1.66	1.51	1.45‐1.56	1.55	1.51‐1.59	2.18	2.01‐2.38	1.65	1.46‐1.87
	1 (lowest)	1.20	1.16–1.23	1.14	1.11‐1.18	1.29	1.26‐1.33	1.19	1.16‐1.22	1.31	1.22‐1.40	1.21	1.10‐1.35
	2	1.18	1.15‐1.22	1.11	1.08‐1.15	1.24	1.20‐1.27	1.19	1.16‐1.22	1.27	1.18‐1.36	1.19	1.06‐1.32
	3	1.13	1.09‐1.16	1.09	1.05‐1.12	1.22	1.19‐1.26	1.14	1.12‐1.17	1.18	1.10‐1.27	1.14	1.02‐1.27
	4	1.04	1.01‐1.07	1.04	1.01‐1.07	1.08	1.05‐1.11	1.09	1.06‐1.11	1.18	1.10‐1.26	0.94	0.84‐1.05
	5 (highest)	1.00	Reference	1.00	Reference	1.00	Reference	1.00	Reference	1.00	Reference	1.00	Reference
**Surveillance, Epidemiology, and End Results stage**													
	Localized	1.00	Reference	1.00	Reference	1.00	Reference	1.00	Reference	1.00	Reference	1.00	Reference
	Regional	4.55	4.44‐4.67	1.73	1.68‐1.78	2.85	2.78‐2.92	2.13	2.08‐2.19	3.06	2.89‐3.24	3.40	3.11–3.72
	Distant	25.43	24.76‐26.11	10.75	10.45‐11.06	6.67	6.50‐6.85	6.04	5.90‐6.19	26.57	25.05‐28.18	14.47	13.17‐15.90
	Unknown	7.64	7.38‐7.92	4.63	4.45‐4.81	2.35	2.29‐2.42	3.52	3.41‐3.64	5.66	5.16‐6.21	4.43	3.93‐5.00
**Deprivation index**													
	1 (most deprived)	1.13	1.10‐1.17	1.13	1.09‐1.17	1.08	1.05‐1.12	1.19	1.16‐1.22	1.19	1.09‐1.31	1.16	1.03‐1.31
	2	1.06	1.04‐1.09	1.06	1.03‐1.09	1.04	1.01‐1.07	1.12	1.10‐1.15	1.10	1.03‐1.17	1.12	1.02‐1.23
	3	1.02	0.99‐1.04	1.04	1.02‐1.07	1.03	1.01‐1.05	1.05	1.03‐1.06	1.02	0.98‐1.08	1.16	1.07‐1.25
	4 (least deprived)	1.00	Reference	1.00	Reference	1.00	Reference	1.00	Reference	1.00	Reference	1.00	Reference

a Not applicable.

## Discussion

### Principal Results

The survival rates for the six different types of cancer were as follows: stomach cancer, 69.6% (96,404/138,462); colorectal cancer, 66.6% (83,406/125,156); liver cancer, 33.7% (23,860/70,712); lung cancer, 30.4% (33,203/109,116); breast cancer, 91.5% (90,730/99,159); and cervical cancer, 78% (12,930/16,580). Our results show a trend similar to the 5-year survival rates in South Korea from 2017 to 2021 [9]. Compared with the 5-year survival rates from 2001 to 2005, stomach, liver, lung, breast, and cervical cancers had higher survival rates, whereas colorectal cancer had a lower survival rate [9]. However, notably, these differences may be influenced by methodological variations in survival rate calculation; thus, direct comparisons may have limitations.

Previous studies have confirmed that these survival rates are influenced by socioeconomic factors, such as income level [10-17]. In this study, we also observed a trend wherein lower income levels were associated with a higher risk of mortality for all six cancer types. This emphasizes the reduced survival prospects for low-income patients following a cancer diagnosis, with a more pronounced pattern compared with the highest-income and medical aid groups.

Interestingly, for breast cancer, which has a high survival rate, the effects of low income on the risk of death due to cancer were most pronounced. In contrast, for cancer types with lower survival rates, such as liver and lung cancers, income levels had a smaller influence. These results support those of previous studies indicating that income-related factors may have a greater effect on the survival of less fatal cancer types [11,39,40]. Similar trends were observed in studies conducted in the United States, where for preventable cancers such as cervical cancer, the mortality rate among low-income individuals was twice as high as that among high-income individuals. However, for high-mortality cancer types, such as liver and lung cancers, the differences were relatively small [11].

Furthermore, as the deprivation index increased, the risk of death also increased. This trend has also been observed in studies conducted in Japan and the United States [23,24]. However, this finding may differ from that of domestic studies. In a study focusing on the Gwangju-Jeonnam region in South Korea, no significant correlation was found between the deprivation index and cancer survival rates [25]. These divergent results may be owing to the fact that this study used a deprivation index at the municipal level for the entire country of South Korea, whereas Kang et al’s [25] study was limited to a specific region. Considering the limited number of previous studies analyzing the relationship between regional deprivation and survival rates, further studies are required to obtain clearer and more consistent results.

The diagnostic stage is recognized as a crucial factor in explaining survival disparities [12,26-28,41], as confirmed in this study. When cancer was diagnosed at an advanced stage, a higher risk of death was observed. Although numerous countries have conducted studies on this topic, the relationship between the disease stage at diagnosis and survival in the context of SES is still not fully understood. A study in the United States has suggested that lower SES leads to delayed diagnosis and less aggressive treatment, resulting in an increased risk of cancer-related death [29]. However, a large-scale study in Canada did not find a strong association between diagnostic stage and survival disparities across SES groups [42]. These variations may be attributed to differences in health care systems and health insurance policies that affect access to cancer screening and care [42].

Our study confirmed that cancer survival rates decrease in conjunction with low SES factors. In particular, income level was prominent, especially in less fatal cancers such as breast cancer. Furthermore, the risk of death increased when cancer was diagnosed at an advanced stage. This emphasizes the importance of early cancer detection, especially in socioeconomically vulnerable populations. In 1999, the South Korean government established the NCSP with the aim of offering organized cancer screening to alleviate the burden of cancer [4]. Since then, the target population and types of cancer included in the NCSP have expanded, and it has been shown that the NCSP is effective in reducing cancer mortality rates [5-8]. Korea has experienced a consistent increase in cancer screening rates since the initiation of mass screening programs [4]. However, socioeconomic disparities remain a major cause of inequality, affecting participation in cancer screening services in Korea [43]. Therefore, to increase cancer survival rates among socioeconomically vulnerable patients, interventions in basic health care and cancer screening programs should be expanded, considering the characteristics of each cancer type. This can help to increase early cancer detection rates and provide appropriate treatment at the right time.

### Limitations

Our study has some limitations. First, the analysis was limited to all-cause mortality. This limitation raises the possibility that differences in survival rates related to income levels may be attributed to differences in mortality rates due to causes other than cancer, potentially leading to an overestimation of the differences in cancer survival rates. Therefore, comprehensive data including cause-specific mortality rates related to income are required. Second, there were difficulties collecting actual income data; therefore, insurance premiums were used as proxies for income. For workplace subscribers, premiums are levied based on their salaries, whereas for local subscribers, premiums are assessed based on various sources of income, leading to potential differences in income levels between the groups. However, no other measure was considered superior as a proxy for real income because health insurance premiums within the 20th decile had no major problem representing actual income levels [44]. Third, we were unable to adjust for all potential confounding factors that could influence survival (eg, psychosocial and medical factors). Therefore, caution should be exercised when interpreting the HRs evaluated using the Cox proportional hazards model.

### Conclusions

In summary, lower income levels are associated with a higher risk of cancer mortality. This effect is particularly pronounced in less fatal cancers such as breast cancer. Additionally, the deprivation index and stage of diagnosis influence the risk of cancer mortality. Therefore, considering the characteristics of each cancer type and socioeconomic factors, it is essential to improve access to basic health care and interventions in cancer screening programs to increase early detection rates and provide appropriate treatment to socioeconomically vulnerable populations.

## Abbreviations

HR: hazard ratio
KCCR: Korea Central Cancer Registry
NCSP: National Cancer Screening Program
NHID: National Health Information Database
NHIS: National Health Insurance Service
SEER: Surveillance, Epidemiology, and End Results
SES: socioeconomic status
